# Management of pediatric ankle fractures: comparison of biodegradable PLGA implants with traditional metal screws

**DOI:** 10.3389/fped.2024.1410750

**Published:** 2024-10-30

**Authors:** Hermann Nudelman, Aba Lőrincz, Anna Gabriella Lamberti, Marcell Varga, Tamás Kassai, Gergő Józsa

**Affiliations:** ^1^ Division of Surgery, Traumatology and Otorhinolaryngology, Department of Paediatrics, Clinical Complex, University of Pécs, Pécs, Hungary; ^2^ Department of Thermophysiology, Institute for Translational Medicine, Medical School, University of Pécs, Pécs, Hungary; ^3^ Department of Pediatric Traumatology, Péterfy Hospital, Manninger Jenő National Trauma Center, Budapest, Hungary

**Keywords:** pediatric, ankle, fracture, PLGA, osteosynthesis, absorbable, biodegradable

## Abstract

**Introduction:**

The relevance of biodegradable implants has gained more importance in modern clinical practice. The study aimed to evaluate the effects and outcomes of ankle fracture treatment with absorbable implants compared to metal screws. These implants are made from poly l-lactic-co-glycolic acid (PLGA), however, there are several other materials available on the market.

**Methods:**

In a retrospective review, a total of 128 patients were under observation, with distal tibial fracture types ranging from Salter-Harris II-IV. In the absorbable group, patients were treated with the implants (*n* = 76). The metal group included patients treated with titanium or steel screws (*n* = 52). The extremities were placed in a cast for six weeks after surgery and were utilized for another 6–8 weeks. Patients were followed up for 12–30 months and were evaluated accordingly. The authors examined several aspects such as age, gender, open or closed repair, mechanism of injury, length of hospitalization, type of fracture, time of recovery, and complications.

**Results:**

There were no statistically significant differences between the groups regarding demographic qualities, such as age, type of fracture, side of injury, and length of cast application (*p* > 0.05 in all cases). Out of 76 patients in the PLGA group, only two presented with complications, so reoperation took place. The rest healed without complications or refractures. Two of those treated with metal screws (*n* = 52) had minor, and four had major complications with reoperation.

**Discussion:**

In pediatric cases, PLGA implants may present excellent results for treating ankle fractures. They do not disturb the growth plate and do not require reoperation. For this reason, they reduce the burden on the patient and the healthcare provider while simultaneously decreasing the risk of complications, such as infections or problems due to general anesthesia.

## 1 Introduction

The ankle ranks as the second most frequent site of fractures that involves the growth plate in children (1, 2). Distal tibial and fibular growth plate injuries constitute 15%–25% of all physeal fractures, with sports activities being the primary cause (2, 3). These include hockey, football and gymnastics which are the most common cause of injury, as well as skating, biking, and skiing. Falls from heights such as trees, horses, or playground equipment constitute a large part in sustaining the injury. These injuries are typically observed in the ages of 8–15, with a greater incidence among boys and children with a higher body-mass index (BMI) (2, 4, 5). This is due to the greater load on the joint the injury could be the result of direct or indirect force. In the pediatric population, injuries that would cause a sprain in adulthood are likely to cause physeal injuries due to the robust structure of the ligaments. Twisting of the ankle is the primary mechanism of injury, leading to supination or pronation (2). Among indirect injuries, injury by supination is ten times more common than pronation. Additionally, pediatric ankle fractures can result from high-energy trauma, such as axial compression seen in falls from a height or crush injuries (1, 2, 6, 7). In younger children, the differential diagnosis should entail osteomyelitis, bone and metabolic disorders, and tumours. Furthermore, below the age of one in children who have not yet learned to walk, child abuse should be considered. Non-accidental trauma can manifest as “metaphyseal corner fractures” of the tibia (1, 2, 8–10).

Children who sustain ankle fractures typically experience an immediate onset of pain, which intensifies with weight-bearing on the affected limb. Moreover, there may be swelling and bruising on the skin, with the extent of symptoms often corresponding to the severity of the injury. Notable displacement of the fractured bone can yield a visible deformity and may potentially disrupt the blood supply to the foot. Therefore, examining both major pulses, namely the tibialis posterior and dorsalis pedis, is vital while assessing capillary refill (11, 12). A comprehensive neurological examination should be conducted on the lower extremity below the injury site, which involves evaluating superficial sensation on the foot's dorsal and plantar aspects. Rare complications involve compartment syndrome, which is due to increased pressure and can impair blood flow, and extensor retinaculum syndrome. The latter leads to pain that is disproportionate to the injury, hypo- or anaesthesia in the first web space, weakness in the toe extensors, and pain upon passive flexion (11, 13, 14). These are ascribable to the excessive transition of bone fragments that lead to compression of structures in the anterior aspect of the extremity, primarily affecting the deep peroneal nerve. The extensor retinaculum is a thick continuation of the anterior fascia of the leg which restrains the tendons at the ankle. Fracture at this site with anterior displacement will compress the contents of the tunnel created by the fascia (13–15).

### 1.1 Classification

**Figure 1:**
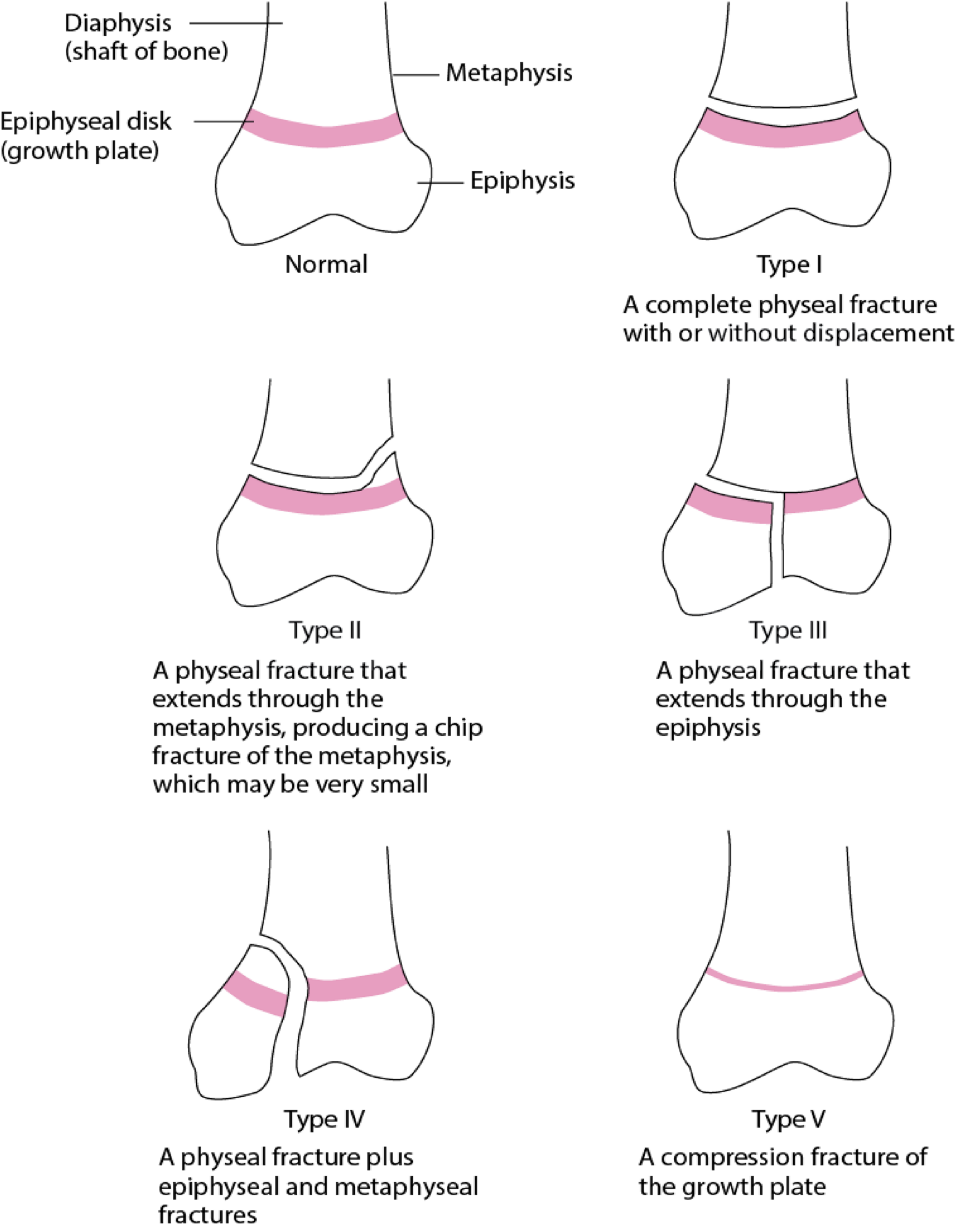
This schematic diagram describes the types of physeal fractures classified by Salter-Harris (17).

A widely adopted system for pediatric ankle fractures is the well-established Salter-Harris Classification (16). Type I fracture involves a direct extension through the growth plate, separating the epiphysis and the metaphysis (Figure 1). As initially described, this type of fracture mainly affects younger children with a thicker physis. Type II is the most prevalent among physeal fractures, constituting approximately 74% of the cases (3, 16). In this type of injury, the fracture line originates along the plane of the physis and then exits through the metaphysis to form what is known as the Thurston-Holland fragment. Salter-Harris type III fractures enter the physis; however, the fracture line exists through the epiphysis, leading to a fracture that involves the articular surface. While these injuries are less frequent, they carry the risk of potential complications such as post-traumatic arthritis or growth arrest (18). Type IV fractures traverse the physis and extend through the epi- and metaphysis (Figure 1). Longitudinal instability arises as both the articular surface and the physis are compromised. The risk of complete physeal arrest and formation of transphyseal bony bars arise due to malreduction, steering to asymmetric growth and deformity. Salter-Harris Type V fractures are characterised by crush injuries at the physis due to compression (Figure 1). They manifest as stress injuries and can be observed in gymnasts who experience repetitive loading with extension. However, these fractures are rare (16).

The growth plate, which stretches from the epi- to the metaphysis, contains four distinct zones: the layer of reserve cells, the proliferative layer, the layer of hypertrophy, and the layer of provisional calcification (19, 20). A decrease in the ratio of cells to matrix hinders the mechanical strength of these structures (19). Fractures typically occur within the hypertrophic zone, characterised by its larger cell and lower matrix content. The layer of reserve cells holds progenitor cells, driving fractures that traverse the physis toward the epiphysis [Salter-Harris (SH) III and IV] and involve the reserve cells more likely to disrupt growth plate development (16, 19, 21). Ligamentous structures, both lateral and medial, insert distal to the physis, increasing the chances of growth plate injury over ligament failure due to the plate's vulnerability to tensile forces and the strength of ligaments.

**Figure 2:**
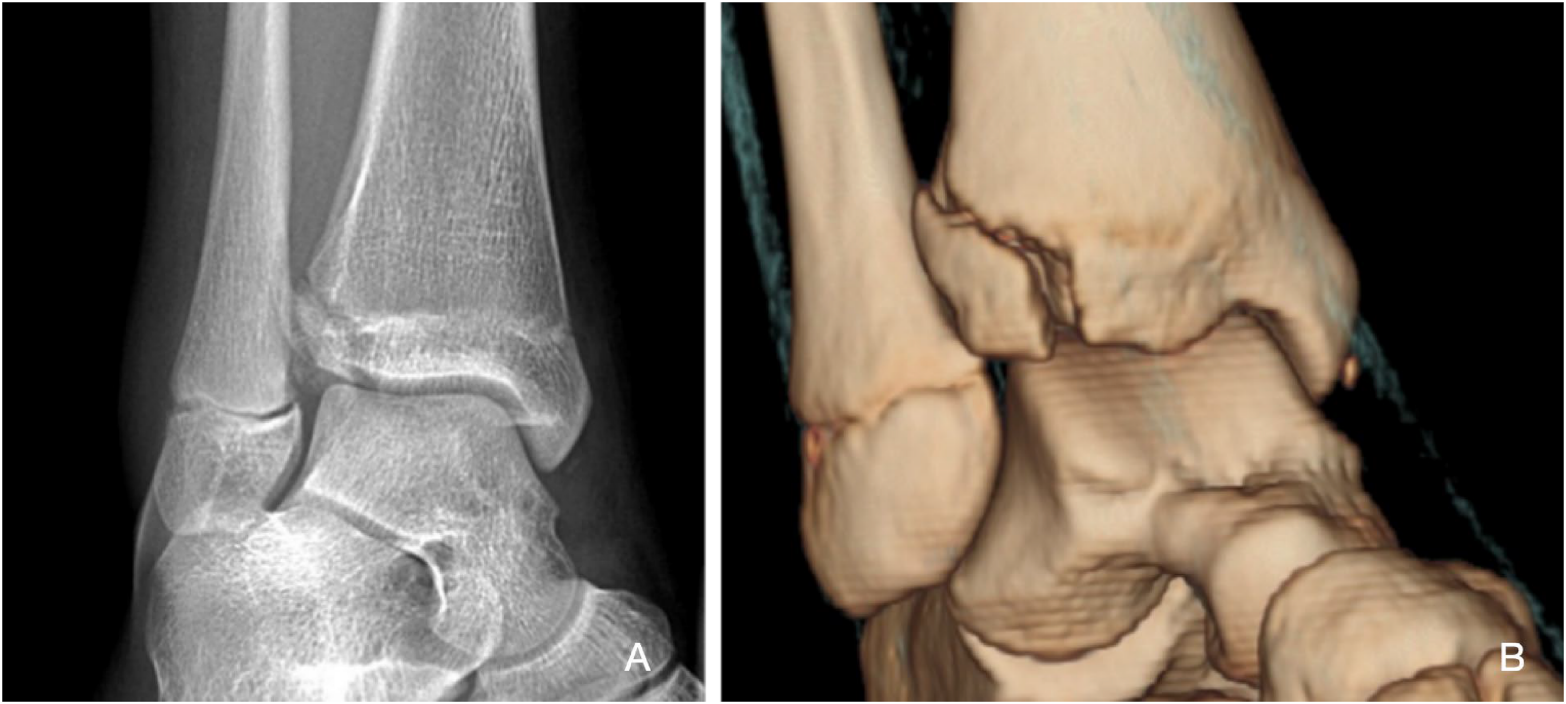
Preoperative x-ray **(A)** and CT scan **(B)** of a Tillaux fracture.

Nonetheless, individuals with a closing growth plate aged between 12 and 15 years are susceptible to a specific SH-III fracture pattern, referred to as a Tillaux fracture (Figures 1, 2). This pattern applies to the anterolateral avulsion of the distal tibial epiphysis at the insertion site of the anterior tibiofibular ligament (ATFL). Triplane fractures are complex SH-IV fractures in younger children and adolescents (1, 2, 6, 16). These fractures affect the metaphysis, physis, and epiphysis, exhibiting horizontal and vertical fracture lines, resulting in multiple fragments; where, as the name suggests, fractures manifest in three distinct planes.

### 1.2 Patient evaluation, diagnosis, and management

Three-view radiographs are obtained on a routine basis. They include anteroposterior, lateral, and mortise views with the ankle in a neutral position. Radiographs should be carefully evaluated at the appearance of the physis. It is important to rule out widening, which is the abnormal increase of space between the bones of the ankle, and syndesmotic injury, which occurs when the fibrous joint holding the distal tibia and fibula together is damaged (12). These hold clinical significance as syndesmotic injury can lead to widening, causing ankle instability and further leads to long-term complications.

Computed tomography (CT) scans are highly suggested in all cases where the articular surface is affected. This aims to choose the appropriate management strategy before entering the operating room, it is a crucial tool for surgical planning for example in the case of Tillaux fractures (Figure 2) (22). This will help to choose a proper treatment strategy in advance, to preserve resources and time. This will aid in avoiding multiple tries at fixation in the operating room, thereby increasing the efficiency of the procedure and the management strategy as a whole.

In particular, when faced with fractures affecting the articular surface, computed tomography (CT) scans offer crucial insights and have demonstrated the potential to alter surgical strategies and can be used in surgical planning. Magnetic resonance imaging (MRI) can provide additional information regarding soft tissue structures and cartilage conditions (1, 11, 20).

When deciding on the appropriate treatment for pediatric ankle fractures, several factors come into play, with particular attention given to the presence of the physis. Children who have experienced an ankle fracture and have more than three years of growth remaining should be monitored continuously for growth arrest. Most treatment options hinge on the condition of the distal tibial component and the status of the syndesmosis (1, 11). The primary objective of management is to restore joint congruency, lower extremity alignment, ankle mortise, and physeal alignment. Preserving the pain-free function of the ankle is paramount (1, 11).

Non-displaced fractures, regardless of their classification, are typically managed conservatively with proper immobilization by cast and protected weight-bearing. It is advised to perform serial radiographs at intervals of 1 week for the first three weeks of treatment to ensure there is no late displacement while in the cast. Generally, a cast treatment will conclude in 4–6 weeks, depending on the patient's age and type of fracture (1, 11, 23).

The initial approach for displaced fractures should involve an attempt at closed reduction, typically carried out under conscious sedation. However, it is essential to note that repeated attempts could potentially harm the physis; therefore, it is advisable not to exceed two attempts (11, 23). If the reduction is deemed satisfactory, the patient is discharged with no body-weight-bearing and elevation precautions. After one week, the control radiograph is taken, and the cast is rebandaged. Control is continuous every week until week four, at which point the patient can transition into a walking cast, allowing for gradual weight-bearing. Ankle ROM exercises are utilized to aid recovery and rehabilitation (1). Activities are encouraged, and a return to sports can occur in most cases after 3–4 months. If the radiographs taken after reduction reveal improper alignment, namely more than 5° of varus or valgus or more than 10° of anterior or posterior angulation (ante/recurvatum) as well as physeal widening of 2–3 mm, surgical intervention should be considered (11, 23). Physeal widening requires an open approach as opposed to malalignment, which could be addressed by closed reduction and percutaneous fixation. CT images are beneficial in this regard, as periosteal entrapment is common in widening (6, 23).

Simple displaced ankle fractures can often be effectively managed with closed reduction (CR) and casting. However, in cases where the fracture pattern in unstable percutaneous fixation or open reduction may be necessary (1). For additional rational stability and to prevent displacement following reduction, a long leg cast with a flexed knee may be employed to ensure the fracture remains in the desired position. Open reduction and internal fixation (ORIF) or mini-ORIF are typically recommended for displaced intraarticular fractures. Internal fixation is achieved by using partially threaded cannulated screws (1, 11).

In some cases, with extensive fracture patterns, plate-screw osteosynthesis might be necessary for optimal stabilization. Whenever feasible, percutaneous fixation with screws and pins is preferred (23). It is paramount to exercise caution when using implants that cross the physis in skeletally immature patients, as they can potentially lead to growth arrest. Minimizing the involvement of the physis should be kept in mind to help prevent growth-related complications. In cases where fixation across is unavoidable in children with open growth plates, it is advised to use smooth pins rather than screws or threaded wires (24). However, if the geometry of the fracture calls for a transphyseal implant, it should be placed perpendicular to the growth plate to ensure minimal disturbance. Metal implants are preferred in many hospitals for their easy use and cost efficiency. Regardless, bioabsorbable screws eliminate the need for removal and might have less impact on articular pressure compared to metal screws (25, 26).

In the case of complex physeal fractures, management often involves six weeks of immobilization and no weight-bearing. Open reduction may be necessary if there is periosteal entrapment or obstruction (11, 23, 24). For SH-I and II fractures, CR might be successful. For SH-III and IV injuries, the optimal approach is anatomic reduction, internal fixation, and joint surface repair. Surgical fixation leads to lower rates of physeal arrest when compared to CR (24).

However, it is vital to proceed with care during an open approach to prevent periosteal stripping at the physis, as this could lead to premature physeal arrest, disrupting standard growth. Triplane and Tillaux fractures may be initially managed with CR. For triplane fractures, reduction can be executed through axial traction and internal rotation. For Tillaux fractures, reduction involves plantar flexion, internal rotation, and pressure over the fragment (1, 11, 23).

### 1.3 PLGA implants

Osteosynthesis has traditionally relied on the utilization of metal screws. Despite their cost-effectiveness, their employment necessitates a subsequent removal procedure, contributing to prolonged hospital stay, and increased risk of infections or complications, thus amplifying healthcare expenses (27, 28). Over the recent decades, there has been notable progress in the development of biodegradable polymers, including poly-glycolic acid (PGA) and poly-lactic acid (PLA) tailored for medical purposes. These degradable polyesters derive from monomers termed lactide and glycolide (29–31).

The vulnerability of PGA to hydrolysis originates from its molecular arrangement, characterized by aliphatic ester bonds and notable crystallinity. In enzymatic or hydrolytic circumstances, carbonyl group cleavage ensues. PGA undergoes complete degradation within four months, albeit experiencing a decline in mechanical resilience around the six-week mark (30). PGA found widespread application as suture material, implantable devices, and as a component for tissue engineering. However, it does undergo rapid loss of strength, produces acidic by-products, as well as a higher rate of inflammation due to a faster degradation rate (32). PLA stands out as a high-strength polymer distinguished by its lower crystallinity. However, its applications are somewhat restricted due to its inherently hydrophobic nature, resulting in minimal water absorption and slow and gradual degradation. The material holds mechanical integrity much longer due to its hydrophobic nature. It also may lead to an inflammatory response and an acidic micro-environment during degradation. Notably, its mechanical integrity persists for the initial months, with complete degradation taking approximately ten months. Despite these limitations, PLA is used and researched as drug delivery systems, biomedical devices, and for the production of fibres and textiles (32).

Absorbable implants that were used in this study feature PLGA, a copolymer derived from glycolic and L-lactic acid, exhibiting commendable attributes of biodegradability and biocompatibility, with minimal adverse host reactions (25). The copolymerisation process aims to address the challenge of PGA's rapid degradation and PLA's slow or incomplete degradation. Upon hydrolytic absorption of the implant, intermediary products such as glycolic and lactic acid are formed. Subsequently, these compounds undergo further metabolism within the body ultimately yielding carbon dioxide and water, both of which are expelled through exhalation and excretion (29, 30).

The degradation process unfolds primarily via hydrolysis, with non-specific enzyme pathways playing a secondary role in the bioabsorption process. Before implantation, the material displays visual transparency and a degree of malleability. Hydrolysis commences upon implantation and progresses until the sixth month, accompanied by a gradual decline in molecular weight and strength over time. *in vitro* observations reveal a transition in appearance from transparent to whitish, indicative of the appropriate degradation process (29, 32, 33). Distinct mechanical attributes, notably absent in metal implants, encompass the diametric expansion and the longitudinal contraction exhibited by the implant. Furthermore, a noteworthy benefit resided in the bending modulus, closely mirroring that of bone, unlike their metal counterparts. This trait serves to guard the fixation from adverse effects as a result of stress shielding. The implants retain their mechanical integrity and resilience for eight weeks, ultimately undergoing complete absorption within two years approximately (33–35).

**Figure 3:**
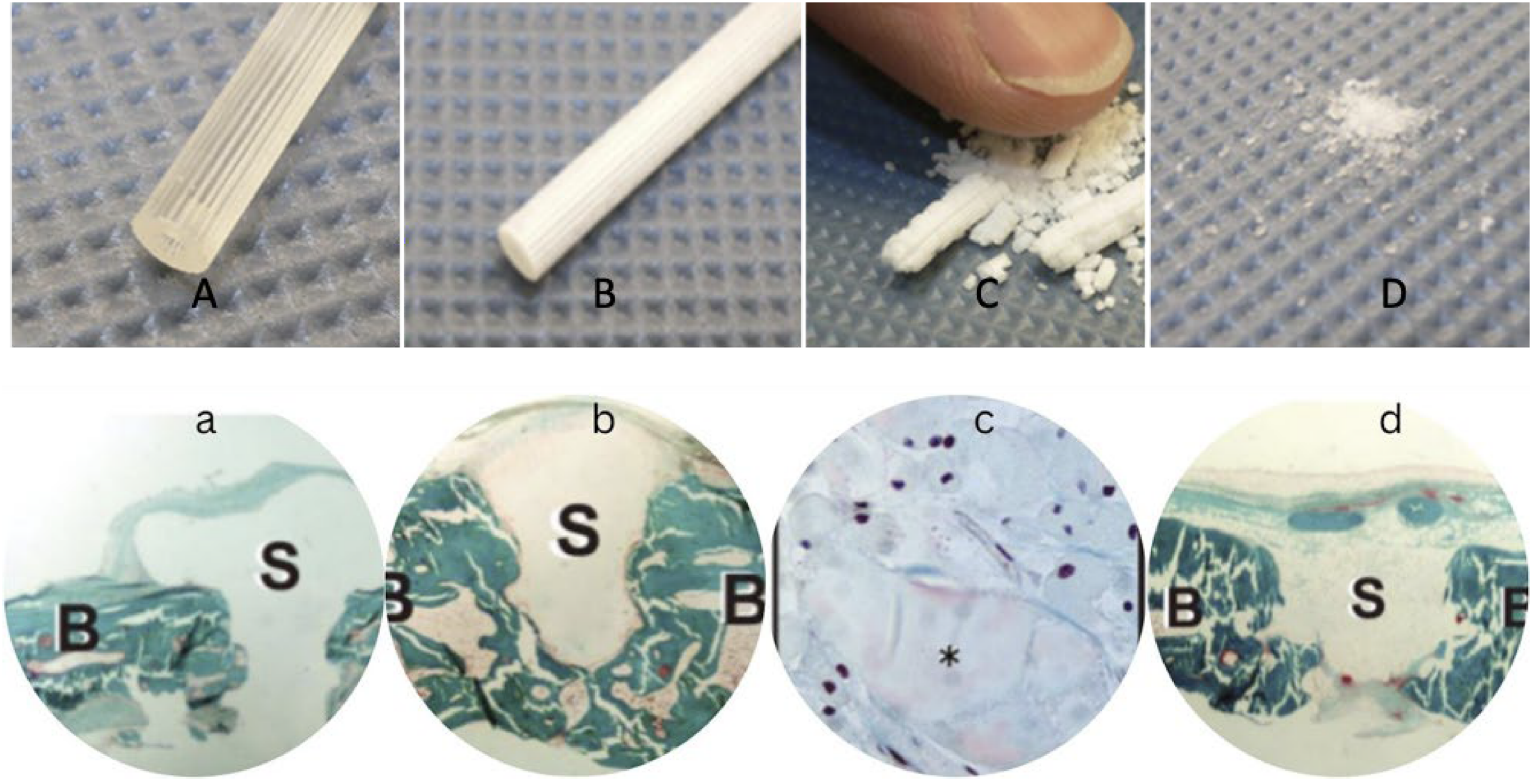
Implants before hydrolysis **(A)**, after six months **(B)**, one year **(C)**, and after two years *in vitro* hydrolysis **(D)** Histological slides (Masson-Goldner trichrome staining) right after implantation in rabbit cranium **(a)**, after 6 months *in vivo* hydrolysis **(b)**, one year -magnified- with prominent osteoblast activity in the vicinity **(c)**, and after two years *in vivo* **(d)**, where B marks the bone while S marks the screws (33–35).

After six months, the implant retains its solidity; however, fragments can be broken off with substantial force. After one year, the remnant of the implants can be broken by applying pressure of a finger-tip, and after two years only a granule-like powder remains (Figure 3). *In vivo*, a fortnight after being implanted into the rabbit cranium, histological analysis reveals modest microvascularization, along with fibroblast and osteoblast activity at the perimeters. By the 24th week, fragmentation can be seen and is accompanied by notable osteoblast activity with macrophages in the surroundings (Figure 3) (25, 30, 33–35).

This paper is based on a retrospective study, which evaluated and compared the results of using PLGA implants and metal screws in pediatric ankle fractures. The authors examined several aspects such as age, gender, open or closed repair, mechanism of injury, length of hospitalization, type of fracture, time of recovery, and complications as well. The paper aims to present the surgical results of the two instruments and their long-term outcomes. It was hypothesised that PLGA implants would be as effective as metal screws in achieving similar outcomes.

## 2 Materials and methods

### 2.1 Study design

This retrospective multi-centre cohort study was conducted in accordance with the Strengthening the Reporting of Observational Studies in Epidemiology (STROBE) guidelines. The patients examined in this study were presented to the two Hungarian pediatric trauma centres at the Department of Paediatrics, Division of Paediatric Surgery, Traumatology, Urology and Paediatric Otolaryngology, Medical School, University of Pécs and at Dr Manninger Jenő Baleseti Centre, Péterfy Hospital, Department of Paediatric Traumatology, 1081, Fiumei Street 17, Budapest between January 2019 and November 2022.

The study retrospectively reviewed 128 patients who underwent surgery due to distal tibia fractures. The groups were divided into Bioabsorbable and metal screws. All patients in the PLGA group (*n* = 76) were treated with biodegradable implants made from PLGA (ActivaScrew™ Cannulated and ActivaPins™ by Bioretec**®**) in an 85l/15G ratio. The cannulated partially threaded implants are available in 3.5, 4.0, and 4.5 mm diameters and 20–90 mm lengths. Pins are available in diameters of 1.5, 2.0, 2.7, and 3.2 mm and lengths of 20–70 mm. Children in the Metal group (*n* = 52) received Kirschner wires and metal (Steel or Titanium) partially threaded screws. Geometries for metal screws are 3.5 or 4.5 mm in diameter and lengths range from 20 to 90 mm, while K-wires are available in 0.6, 0.8, 1.0, 1.2, 1.5, and 2 mm diameters from 100 to 300 mm lengths.

### 2.2 Inclusion and exclusion criteria

(1) Children (<18 years old) with (2) open growth plates who fractured their (3) distal tibia, which was (4) classified by Salter-Harris were included. Children with (1) hereditary or (2) acquired bone disease and (3) closed growth plates were excluded.

### 2.3 Variables and data measurement

The procedures were performed under general anaesthesia with antibiotic prophylaxis, as is used routinely. In all cases, the surgery was done by surgeons with experience in pediatric trauma surgery. In the PLGA group, absorbable implants were used, either 1 or 2 or more, depending on the need for stabilization. In the Metal group, the correction was done using the metal screws. The implants were not in contact with the growth plates, and their insertion was proximal or distal to the physis. Data measurement took place by physical examinations, classification by Salter-Harris based on x-ray images, and data collection based on clinical findings and instruments used.

### 2.4 Surgical management

Clinical application of the technique was accepted and permitted in 2010 by our medical review board, the Hungarian Pediatric Trauma Committee, and the Hungarian Paediatric Surgery Committee. The work was performed in Pécs and Budapest at the Surgical Division, Department of Paediatrics, Medical School, University of Pécs, 7 József Attila Street, Pécs, H7623, Hungary and at the Department of Paediatric Traumatology, Péterfy Hospital, Manninger Jenő National Trauma Center, 1081, 17 Fiumei Street, Budapest, Hungary. The study was conducted in accordance with the Declaration of Helsinki and approved by the Institutional Review Board (or Ethics Committee) of the Surgical Division, Department of Paediatrics, Medical School, University of Pécs for studies involving humans.

#### 2.4.1 Reduction

Reduction should be performed for displaced fractures and should take place as soon as possible. Done by applying gentle manipulation to the extremity, avoiding forced reduction or multiple attempts. Insertion of tools into the physis should be avoided as it could lead to injury, which may result in iatrogenic complications (11, 23). Reductions can be closed, arthroscopic, or open. Open or mini-open reduction (<2 cm incision to visualize the fracture gap) is better than multiple attempts of a closed approach (1). It is suggested to use open reduction and internal fixation (ORIF) regarding SH III and IV fractures with a residual gap of >2–3 mm (11, 23, 28). Post-reduction radiographs are to be obtained to assess alignment and confirm physeal reduction.

#### 2.4.2 *De novo* application of PLGA screws

**Figure 4:**
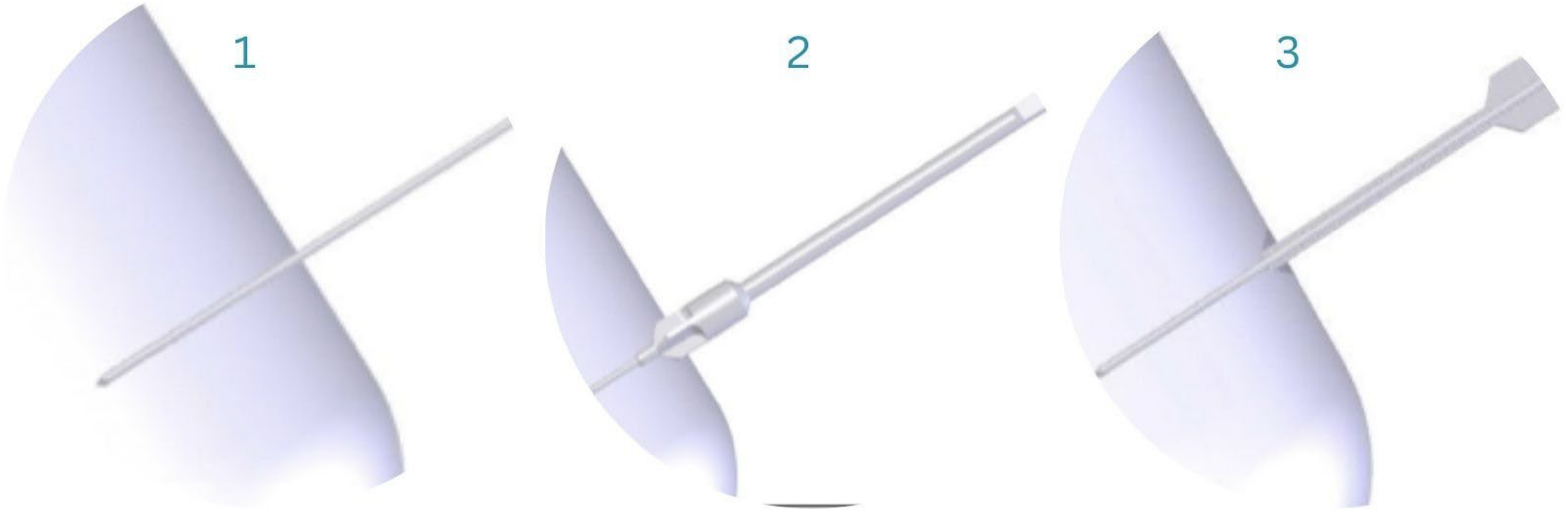
Guide-wire insertion (1), countersink (2), depth gauge to determine screw length (3) (33).

After reduction has been confirmed, the 1.6 mm guide wire is inserted across the fracture, guided by fluoroscopic imaging when needed throughout the surgery. Space for the screw-head is created with the countersink, after which the depth gauge is used to determine the screw length (Figure 4).

**Figure 5:**
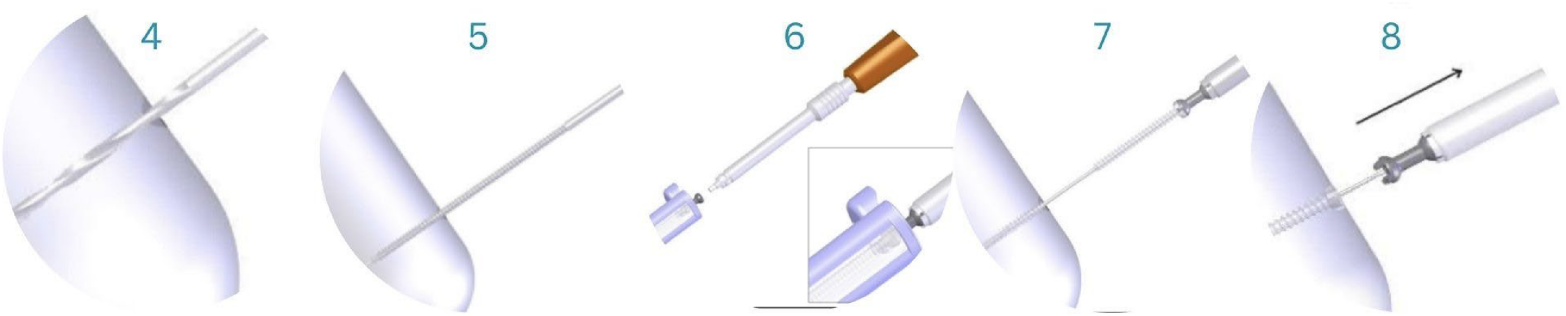
A 3.5 mm screw hole is drilled with a cannulated drill bit (4); after, the entire length of the hole is tapped with a 4.5 mm tap (5). The implant is removed from its container (6), and with the help of a screwdriver, it is inserted along the guide wire (7). Protruding ends of the screws are discarded (8) (33).

A 3.5 mm screw hole is drilled with a cannulated drill bit over the guide wire to a satisfactory depth. The total length of the hole is tapped with a 4.5 mm tap. Then, a screwdriver inserted the 4.5 mm ActivaScrew™ Cannulated along the guide wire. The guide wires and metal screw heads were removed, and the protruding parts of the screws were discarded using high-temperature cautery, a surgical saw or cutting forceps to avoid soft tissue irritation (Figure 5).

#### 2.4.3 General considerations of screw fixation

The Arbeitsgemeinschaft für Osteosynthesefragen (AO) guidelines for physeal fracture repair provide crucial notes that should be followed when dealing with injuries affecting the growth plates (24). The most important points are noted to achieve proper fixation and healing and to guide surgical principles (24, 36):

• After proper reduction
• With the use of cannulated, partially threaded screws
• Perpendicular to the fracture line
• From the direction of the broken fragment
• Primary care for the articular component
• Do not cross the growth plate
• Do not use fully threaded or non-threaded screws

Screws are placed parallel below or above the physis when applying an anteromedial approach. The implants should not be in contact with the growth plate as it could lead to growth disturbance. However, if the geometry of the fracture calls for a transphyseal implant, it should be placed perpendicular to the growth plate to ensure minimal disturbance (1, 6, 24). The metal screw was put in place by utilising the standard technique. After preparing the bone, a guidewire was inserted into the desired location for the screw. A cannulated drill was then used to create a pathway for the screw. The metal screw was inserted over the guidewire, ensuring stable fixation of the graft. The screw was advanced until it reaches the opposing cortical surface to avoid protrusion into the joint space. The stability of the fixation was confirmed through manual testing before closing the incision.

#### 2.4.4 SH-II fracture fixation

**Figure 6:**
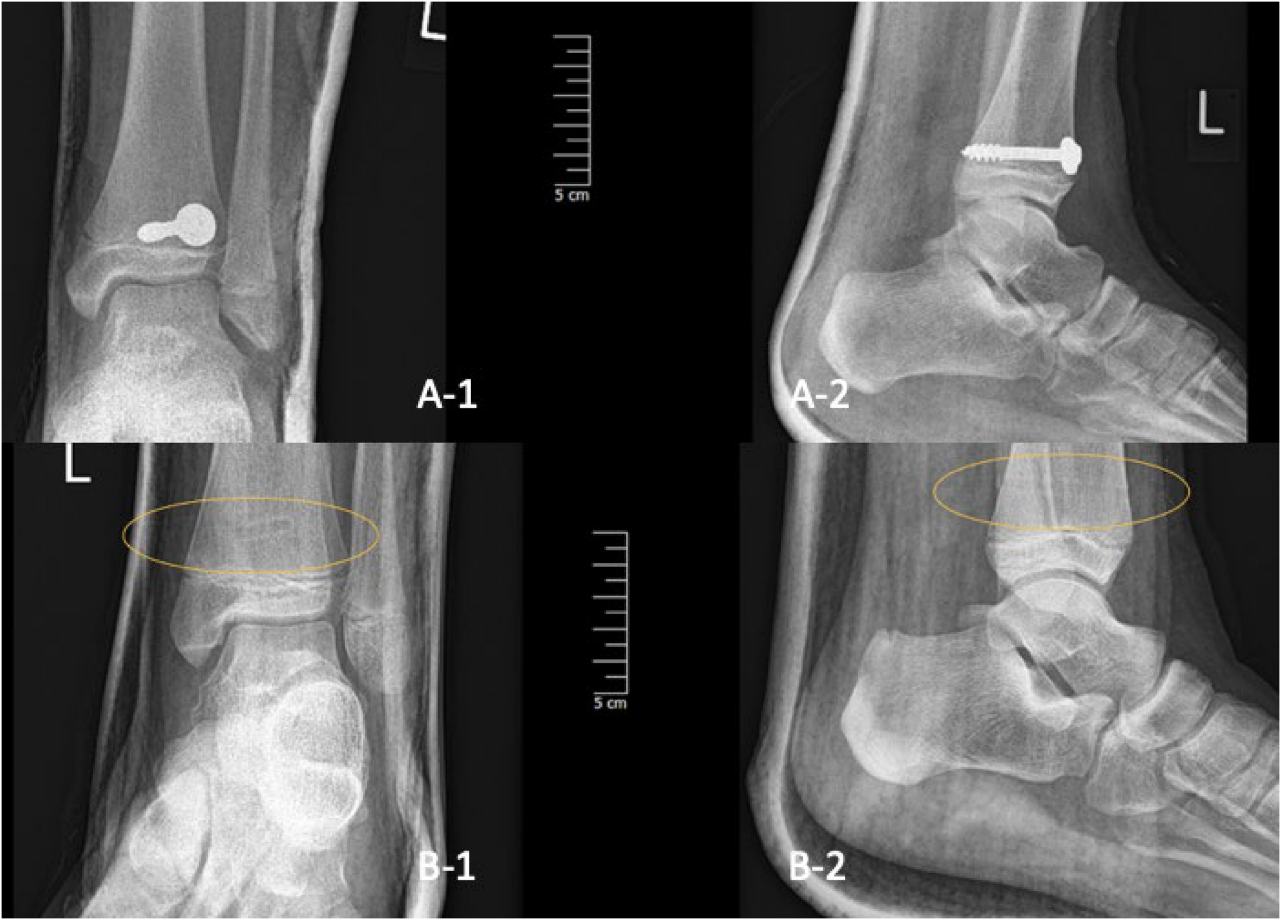
Two-plane radiographs of SH-II fracture one-day post-op in one child treated with metal (A-1/2) and in another treated with PLGA implants (B-1/2), the implant is marked with the yellow oval.

These are addressed by employing a lag screw through the metaphyseal (Thurstan Holland) fragment towards the metaphysis of the proximal fragment, aligned parallel to the physis. If the piece is of sufficient size, two screws may be implanted. The fragment may be situated posteriorly, posterolaterally, or laterally. If rapid reduction of the fragment is achievable, screws can be directly inserted into its side. In the case of a posterior fracture pattern, insertion from anterior to posterior is typical. For a lateral fragment, screws are positioned from posteromedial or lateral anterior to the fibula. Medial soft-tissue damage, calls for lateral insertion (11, 23). The underlying soft tissues are gently dissected and a soft tissue protector is placed on the bone. The lag screw is inserted in the chosen direction aiming for the center of the fragment. Operation is finished only after confirming reduction, fracture stability, and screw placement with fluoroscopy (Figure 6).

#### 2.4.5 SH-III fracture fixation

**Figure 7:**
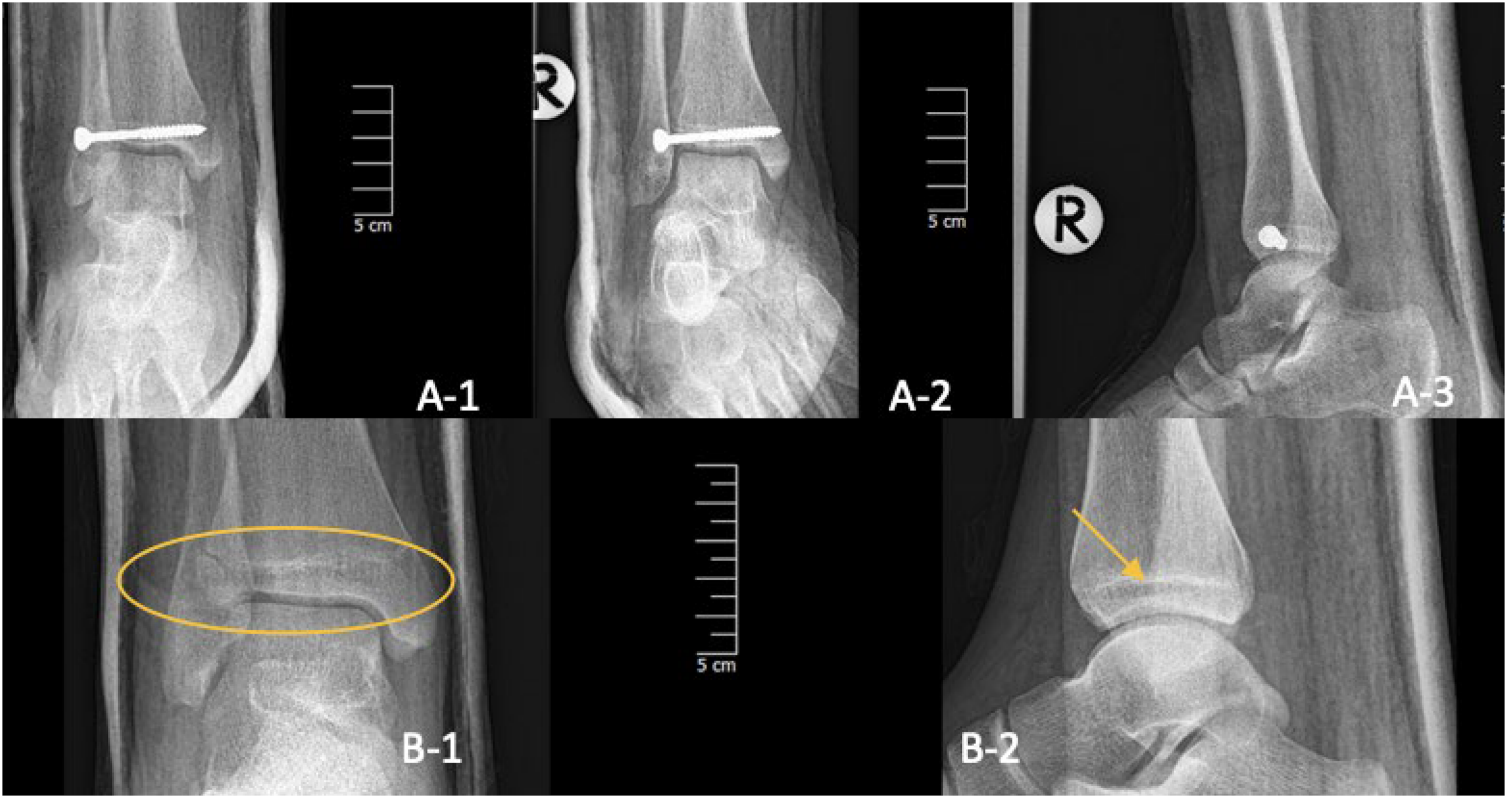
Three-plane radiographs of SH-III fracture (Tillaux) one-day post-op in one child treated with metal (A-1/2/3) and two-plane radiographs in another treated with PLGA implants (B-1/2). The PLGA inserts are marked with a yellow oval and arrow.

Displaced fractures affecting the articular surface demand an open approach with an anatomical reduction to restore the articular congruency and align the physis. Typically, the screws are placed medial-to-lateral, parallel to the physis (Figure 7). Two screws are to be used in large defects. The screws are inserted parallel to the physis. Percutaneous insertion of K-wires and screws can be achieved, however, the reduction is performed through the anteromedial incision. Pay attention to any entrapped periosteum, perform sufficient debridement of nonviable tiny fragments or clots and verify proper alignment by direct vision. Reassess the alignment and implant position both visually and with an x-ray prior to waking. The stability of the fixation is ensured by performing a range of dorsi/plantar flexion (37).

#### 2.4.6 SH-IV fracture fixation

**Figure 8:**
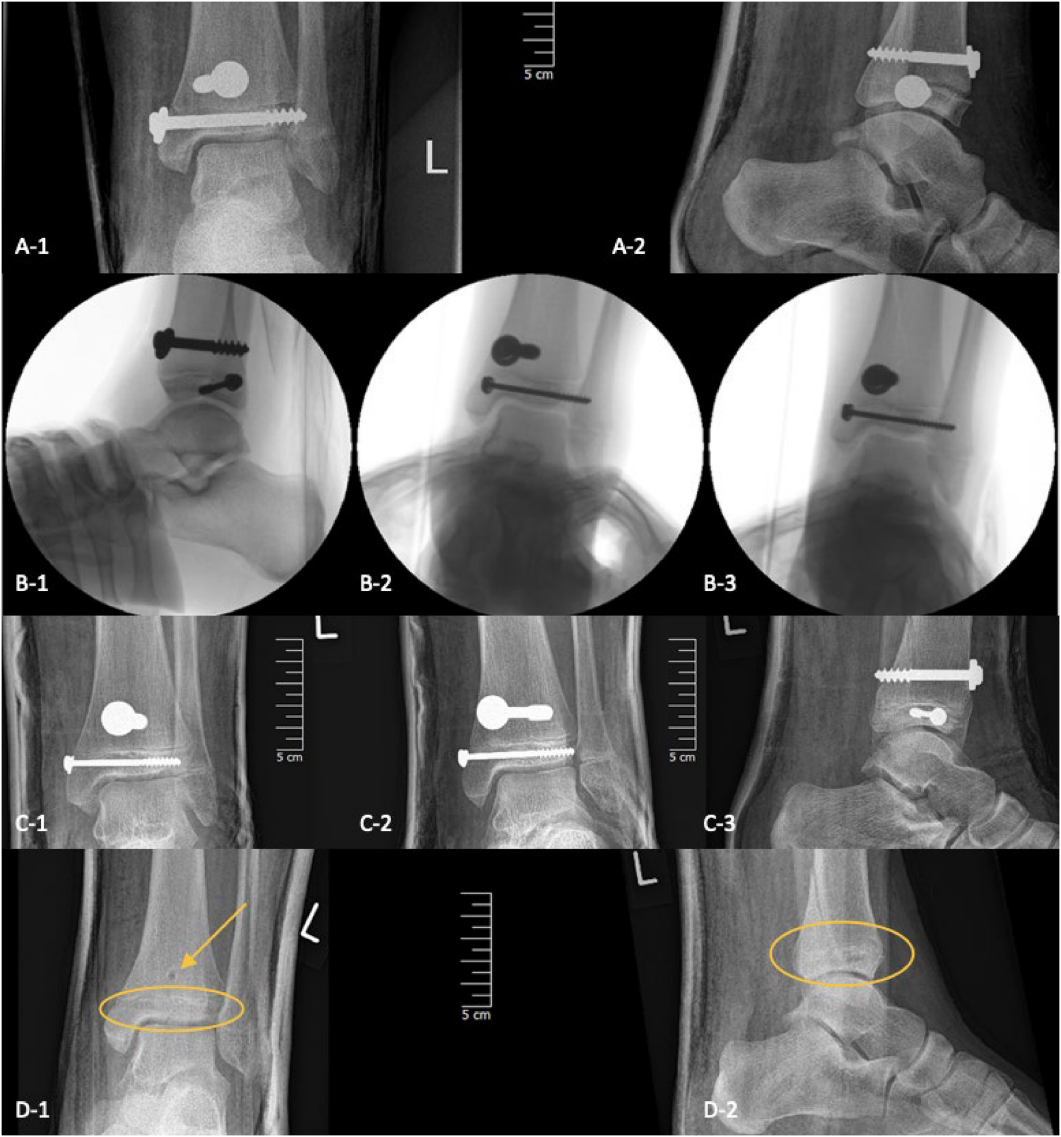
Three-plane radiographs of SH-IV fracture one-day post-op in three children treated with metal (A/B/C-1/2/3) and two-plane radiographs in another treated with PLGA implants (D-1/2), the PLGA implant is marked with the yellow oval and arrow.

In addition to applying a trans-epiphyseal screw, another implant is positioned over the physis, transmetaphyseally. An open approach is recommended in cases of displaced fractures with articular surface involvement. Through an anteromedial course, any blocks to reduction are dismissed and the fracture is stabilized (Figure 8). Periosteal entrapment is to be evaded and the wound site is to be cleared (1). Anatomical reduction should be affirmed by direct vision. If the situation calls for it, K-wires can be used to stabilize the fracture. Screws are inserted parallel to the physis, one in the epiphysis and one in the metaphysis (6, 23). Reassess alignment and the correct position of the implants and evaluate stability by performing a range of motion movements with dorsi/plantar flexion (6, 11, 23). The implants should not be in contact with the growth plate as it could lead to growth disturbance. However, if the geometry of the fracture calls for a transphyseal implant, it should be placed perpendicular to the growth plate to ensure minimal disturbance (1, 6, 21).

#### 2.4.7 Postoperative and follow-up period

Radiographic images were obtained post-operatively and during the third, fourth, and sixth weeks of healing. Cases were followed up to 30 months at most with several control x-ray examinations, which proved unremarkable during healing. Dorsi- and plantarflexion, inversion-eversion, and lateral and medial rotations were examined according to their range of motion (ROM). Functional results were noted only in the case of pathologies, which could not be identified during the healing period. In one case, hospitalization and subsequent care increased to 22 days due to infection and the use of an ex-fixateur, which later set the course towards proper healing. No long-term complications could be identified in either group after the 30th month.

### 2.5 Statistical methods

The systematization, visualization, and statistical analyses were done with Microsoft Excel 2021, Python, and R-4.2.2. Statistical significance was established as a *p*-value of <0.05 with all data and significance values (*p*-values) rounded to the third decimal.

For primary characteristics (e.g., right/left, sex), mechanism of injury, fracture type, need for CT, and location, Chi^2^ Test was used. The number of screws, length of cast application, and age were evaluated by Mann-Whitney *U*-test.

**Figure 9:**
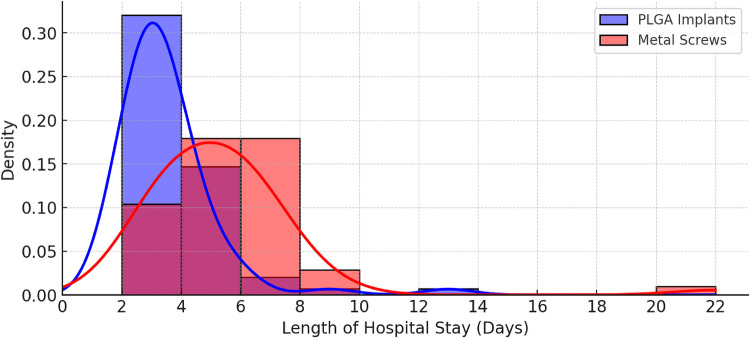
Length of hospital stay in days.

For the evaluation of outcomes (complication rates, minor and major complications, reoperation), open-to-closed approach, and mechanism of injury Chi^2^ Test and Fischer's exact test were used, Cramer's V was calculated to show the strength of the association with a minimum threshold of 0.1 (>0.5 = High association, 0.3–0.5 = Moderate association, 0.1–0.3 Low association, <0.1 = none or negligible), LOS was evaluated using Mann-Whitney *U*-test, which can be seen in Table 1 and Figure 9.

**Table 1:** Summary of statistical tests and their results.

Variable	Test used	*P*-value	Significant
Age (years)	Mann-Whitney *U*	0.793	No
Length of hospital stay (Days)	Mann-Whitney *U*	**<0**.**001**	Yes
Application of cast (weeks)	Mann-Whitney *U*	0.056	No
Number of screws (*n*)	Mann-Whitney *U*	0.755	No
Gender	Chi-squared	**0**.**033**	Yes
Side of injury	Chi-squared	0.472	No
Mechanism of injury	Fischer's	“Fall”: 0.277	No
		“Fall from height”: 1.0	No
		“Hit/Traumatic injury”: 0.701	No
		“Sport injury”: 0.195	No
Fracture type (Salter-Harris)	Chi-squared	**0**.**024**	Yes
CT (1-yes)	Chi-squared	0.454	No
Open approach (1-yes)	Fischer's	0.092	No
Complications (1-yes)	Fischer's	0.061	No
Location	Chi-squared	**0**.**004**	Yes

Bold numerals indicate significant values.

## 3 Results

**Figure 10:**
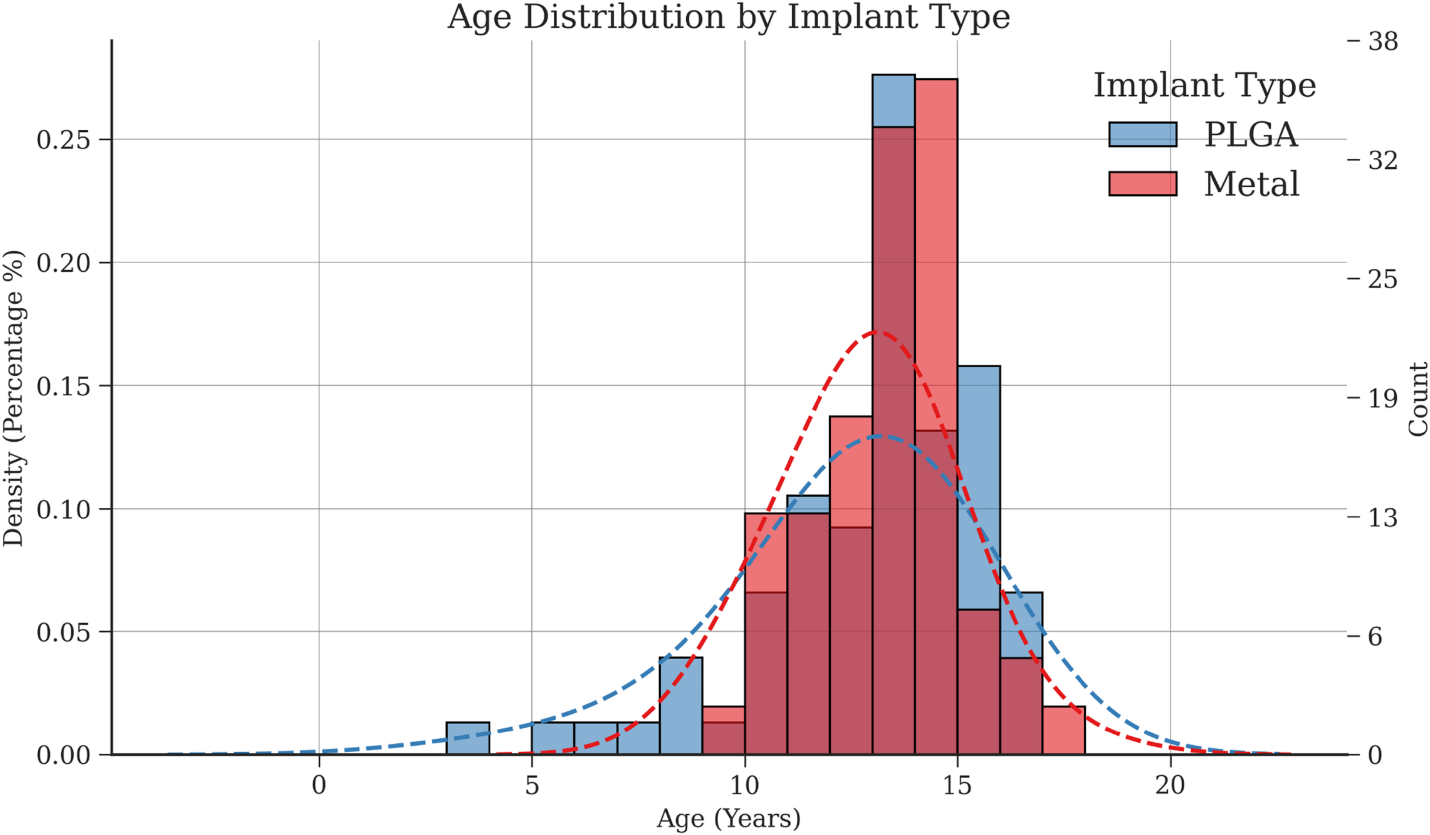
Overlapping histograms of the age distribution.

A total of 128 patients were under observation, with distal tibial fracture types ranging from Salter-Harris II–IV. The mean age was 12 for both groups with a standard deviation (SD) of 2.919 (PLGA) and 1.93 (metal) (Figure 10). In the PLGA group (*n* = 76), 22 SH-II, 28 SH-III and 26 SH-IV fractures were analyzed. This group was composed of 24 boys and 52 girls. The metal group (*n* = 52) contained 26 SH-II, 9 SH-III, and 17 SH-IV fractures and included 25 girls and 27 boys.

There were no statistically significant differences between the groups regarding demographic qualities, such as age, mechanism of injury, side of injury, and length of cast application (Table 1, *p* > 0.05 in all cases). Therefore, the groups were assumed to be homogenous. Numerical visualisation can be seen in Table 1. The Chi test showed statistical significance regarding the implant type and type of fracture, χ^2^(2) = 7.4842, *p* = .024, Cramér's V = 0.2447 (slight association). Further significance was established regarding location χ^2^(1) = 9.305, *p* = .004, Cramér's V = 0.2696 (slight association), and gender.

In one of the clinics, a higher percentage of patients needed mini ORIF when using PLGA implants. A statistically significant difference was not shown regarding this aspect. This distinction is primarily due to material handling, as positioning requires proper sight for the correct angular placement of the implant. Data for the second clinic regarding this information was unavailable.

Sixteen patients were treated with a single screw and eight with two, while one required three and another four screws, respectively. An increase in the number of screws can be explanied by difficult fixation. Fracture geometry might call for additional implants to achieve total fixation, as it is in the case of SH-II fractures when the metaphyseal fragment needs additional stability. Among those treated with metal implants, only three out of 32 were treated with ORIF, showing that the physical manipulation of the instrument is possibly easier. Twenty patients were treated with a single screw, and eleven required two. Data for this variable from one of the clinics was not available, therefore the analysis could not yield trustworthy results.

Hospital stay was a maximum of 13 nights in the PLGA group (SD = 1.505) and 22 nights (SD = 1.150) in the metal group, while the mean was 3.54 and 5.53, respectively. Mann-Whitney *U*-test showed a significant difference between the groups (*p* < 0.05) (Table 1).

The average overall complication rates in the groups were two out of 76 and six out of 52, respectively. Among those treated with PLGA, these were epiphyseal step-formation and screw malposition. In the metal group, one case had to undergo reoperation due to bone necrosis, one because of an adverse host reaction, and screw malposition in two cases. One reoperation included bone fragment removal and another correction due to syndesmolysis.

While we can see a lower rate among those treated with biodegradable implants, no statistically significant difference can be observed. A deep statistical analysis showed a slight association in complication ratios, providing a minor insight into the prospects of care given and the therapy outcome (Cramér's V = 0.1807). However, results are limited by the low incidence rates and the small sample size of the groups.

## 4 Discussion

Our hypothesis postulated that employing PLGA implants would yield comparable outcomes to those achieved with metal screws. Our assumptions were grounded in the following rationales:

1. Avoidance of Second Surgery: Biodegradable screws negate the requirement for secondary surgical intervention, thereby eliminating the need for implant removal (33, 38).
2. Resource Conservation: The absence of an additional surgery translates to a reduction in the consumption of materials and resources to be used. These align with cost-effective and resource-efficient medical practices (33, 38).
3. Decreased Duration of Hospitalisation: Without additional intervention, the period of hospital stay is abbreviated, contributing to more efficient utilization of healthcare facilities and quicker return to normal patient activities (38).
4. Minimized Skin and Soft Tissue Irritation: PLGA implants tend to cause fewer instances of skin and soft tissue irritation, promoting better postoperative comfort and recovery (33, 39).
5. Alleviation of Strain: Eliminating an additional surgery lessens the physical and psychological strains on the patient and the healthcare providers involved.
6. Molecular Restructuring of the Implants: As the implants undergo hydrolysis, they are remodelled on a molecular level. This leads to the diametric expansion which ensures the implant is locked in place effectively. The longitudinal shortening of the implant serves the purpose of firm compression of the fracture line (29, 34, 35).
7. Complete Absorption and bone ingrowth within 2 years: One of the greatest advantages of the implants is that they allow for bone in-growth and remodelling (40, 41).

These considerations collectively formed our hypothesis, suggesting that the use of bioabsorbable PLGA implants could potentially yield equivalent outcomes to those achieved with traditional metal implants. These types of implants offer notable benefits for pediatric patients, as they facilitate bone growth and remodelling without requiring a second implant removal operation. Moreover, they are less likely to induce inflammation or irritation at the implant site, which can be a concern with metal screws. The advantages and disadvantages of both techniques are summarized in their main points in Table 2.

**Table 2:** Summary of the advantages and disadvantages of PLGA and metal implants.

	Advantages of PLGA implants	Disadvantages of PLGA implants	Advantages of metal implants	Disadvantages of metal implants
Primary features	Facilitation of bone growth and remodeling: Biodegradable implants, composed of materials that gradually break down and are assimilated by the body, enable ongoing bone growth and remodeling. This obviates the necessity for a subsequent surgical procedure to extract the implant (33, 40).	Potential for Reduced Strength and Durability: In specific fracture scenarios, especially among active children or those engaged in sports, biodegradable implants might not offer the same level of strength and durability as metal alternatives (42).	Stability: Metal screws offer a dependable framework, a stable construct for the fracture to heal, and can be removed if necessary.	Risk of implant failure, complications and neccesary removal: The associated risk of implant failure and potential complications could necessitate further interventions. As is the case with removal.
Cost		Higher initial cost: Biodegradable implants tend to be more expensive than metal screws and may not be covered by insurance in some cases (33, 43).	Cost-Effectiveness: Metal screws are more economical than biodegradable implants and have a proven track record of successful use over many years with good results (43).	
Radiographic Visibility	Compatible with MR Imaging: The implants can be visualized on MR imaging and therefore the degradation process can be monitored through radiography (33, 40).	Limited Visibility on x-ray: The implants are nearly impossible to identify on x-ray imaging without Tri-Calcium-Phosphate (TCP) tips or inlay, which may complicate postoperative assessments (33, 40).	Radiographic Visibility: Metal implants are easily detectable on radiographs, facilitating straightforward assessment and monitoring.	Incompatibility with MR Imaging: When the implants are incorporated into the patient, an MR examination is impossible.
Interactions	Reduced Risk of Irritation and Inflammation: Biodegradable implants are less prone to trigger irritation or inflammation at the implant site, a concern that may arise with metal screws (42).	Acidic Environment: Degradation by-products may form an acidic environment which decreases osteoblast activity (44, 45).		Potential for Irritation and Inflammation: Metal screws may cause irritation or inflammation at the implant site, which can be a concern in pediatric patients.
Removal	No hardware removal surgery:			Necessary hardware removal.
Access		Limited Availability: Biodegradable implants may not be universally accessible across all medical facilities or clinics, potentially making them harder to procure.	Accessibility: Metal screws are readily available and commonly stocked in most medical institutions, ensuring broad availability.	

PLGA has been used for decades for tissue engineering and drug delivery systems. In the field of bone tissue engineering, it has been utilized due to its high biocompatibility, mechanical characteristics, and most importantly, tunable biodegradability. This controlled absorption results from the ratio of copolymerisation in the implant, with the fastest degradation rate at a 50G/50L ratio (34). Modifying the composition will result in changes in mechanical and molecular properties impacting polymer degradation mostly. Our implants are made from an 85L/15G ratio (33). This allows controlled absorption of the implant, ensuring that degradation unfolds at a steady rate unlike with its predecessors PGA or PLA which degrade too fast or not adequately. The copolymerisation addresses this issue and provides the required strength for the initial weeks of healing. The bioabsorption has been studied by Landes et al. in cranio-maxillary osteosyntheses, based on radiological evaluations, yielding complete absorption and recanalisation by bone within 2 years (41). Another study, conducted by Hedelin et al. investigated the absorption of implants with MR imaging in pediatric hip osteosyntheses, proving that over 90% of the implants were absorbed and the screw canals were filled with bone (40).

Nonetheless, an acidic environment is produced during the degradation process, which may halt osteoblast activity and increase pro-inflammatory cytokines, however, the acidic environment has been shown to increase degradation. This is the result of the formation of lactic and glycolic acid as intermediary by-products, which locally lower the pH. Researchers have produced 3D-printed scaffolds with the addition of TCP or MgOH to counter these effects (46, 47). Further investigations into PLGA scaffolds with ideal pore size and combinations with other materials are being researched and show promising results (29, 46, 47).

The greater need for open reduction can be explained by the difficulty in handling the implant. PLGA implants are more difficult to manipulate during surgery, more strenuous to see, and more challenging to apply, which would explain the need for proper visibility.

It is important to note that biodegradable implants are more costly than metal screws, may not offer the same level of robustness or durability in certain fracture cases, and may not be as readily available (28–30, 48). Open and complicated fractures should be given appropriate care and are potentially managed with the use of metal implants or in difficult cases an ex-fixateur (49). Conversely, metal screws present a more conventional option for treating pediatric ankle fractures, primarily due to their ability to establish a stable framework for fracture healing (1). Still, metal screws may cause irritation or inflammation at the implant site, and they do not facilitate bone growth and remodelling as biodegradable implants do (22, 27, 28, 38, 49). In addition, as can be seen in Figure 10, metal screws were not used in patients younger than 9 years old. In majority of these cases we opted for the PLGA implants mainly to aovid a second surgery and with this better compliance while easing burden. However, these cases arrived at an ideal time when materials were available in abundance.

Complication rates were seemingly lower in the case of PLGA implants, however, the difference was statistically not significant. The statistical analysis yielded a low association between the outcomes and the method that was applied which necessitates further investigations with larger sample sizes. The two complications in the case of PLGA implants were resolved after correction surgery. Among those treated with metal implants, in one case an ex-fixateur was used to stabilize a fracture leading to the most days spent in the hospital by the patient. This case elongated due to the development of an infection which lead to reoperation and the application of the stable external construct.

In summary, both techniques are appropriate for correcting pediatric ankle fractures. However, the decision of which treatment to use should be made individually, considering the case's specifics. Ankle fractures represent a frequent occurrence among children and adolescents. The management of such fractures can profoundly influence a child's future functionality and mobility. Flawed or inadequate repositioning may result in insufficient fixation, potentially causing structural rotation, displacement, or shifting. Metal implants might provide excellent stability and durability but not allow for remodelling or growth. Complicated, open and multiple fractures require greater stability which could be provided by metal implants. However, biodegradable implants have proven to be effective in treating simple and some complex fracture patterns as well with screws, nails, pins, and even intramedullary nails. They are being researched for the fixation of osteochondral fractures in several settings, with some using individualized 3D-printed scaffolds.

Small retrospective studies about pediatric ankle fractures have several restrictions to be considered when interpreting the results. The limitations include the small sample size, the study's retrospective nature, the lack of control for confounding variables and bias, the lack of ability to establish causality, and the limited standardization of the findings. Therefore, additional large-scale and well-designed studies are needed to validate the results of small retrospective studies. Nevertheless, contemplating those mentioned earlier, we firmly believe that treating pediatric ankle fractures with biodegradable fixation is a promising technique with favourable results.

## 5 Conclusions

This study demonstrates that both metal and PLGA implants are equally effective in treating fractures. However, the application of biodegradable implants, such as PLGA, offers significant benefits including a decreased number of days spent in the hospital, elimination of a second surgery, and promotion of bone growth and remodelling. These qualities contribute to a less invasive recovery period for the pediatric population. Patients may recover in the comfort of their homes, while the healthcare provider may focus on other urgent duties and surgeries. Further research into the application of PLGA implants and scaffolds should be conducted, as the material has proven effective in various settings.

## Data Availability

The original contributions presented in the study are included in the article/Supplementary Material, further inquiries can be directed to the corresponding author.
